# The Development of an Ultrasound-Based Scoring System for the Prediction of Interstitial Pregnancy

**DOI:** 10.3390/jcm14124238

**Published:** 2025-06-14

**Authors:** Yun Ji Jung, Hyun-Soo Zhang, Eun Jin Lee, Hayan Kwon, Ja-Young Kwon, Young-Han Kim, JoonHo Lee

**Affiliations:** 1 Department of Obstetrics and Gynecology, Institute of Women’s Medical Life Science, Yonsei University College of Medicine, Seoul 03722, Republic of Korea; ccstty@yuhs.ac (Y.J.J.); ewhamedlej@naver.com (E.J.L.); whitekwon@yuhs.ac (H.K.); jaykwon@yuhs.ac (J.-Y.K.); yhkim522@yuhs.ac (Y.-H.K.); 2 Biostatistics Collaboration Unit, Department of Biomedical Systems Informatics, Yonsei University College of Medicine, Seoul 03722, Republic of Korea; hszhang@yuhs.ac

**Keywords:** clinical decision-making, cornual pregnancy, ectopic pregnancy, interstitial pregnancy, predictive value of tests, ultrasonography

## Abstract

**Background/Objectives**: Diagnosing interstitial pregnancy (IP) using ultrasonography can be challenging, as it is often mistaken for eccentrically located intrauterine pregnancy (IUP). In this retrospective cohort study, we aimed to develop a predictive scoring model using multiple clinical factors to enhance the diagnosis of IP and facilitate timely interventions in suspected cases. **Methods:** We enrolled 63 pregnant women with a diagnosis of suspected IP who visited a single tertiary center between January 2006 and December 2023. Data on the clinical risk factors, symptoms, laboratory test results, and ultrasound findings were analyzed. A statistical predictive score was developed using logistic regression analysis with feature selection based on the least absolute shrinkage and selection operator to optimize the predictive accuracy and clinical applicability. **Results:** From a total of 12 factors, a scoring model was constructed from the three most prominent factors—ultrasound findings showing no surrounding endometrium, myometrial thinning of less than 5 mm, and vaginal bleeding—all of which demonstrated high feature importance. This predictive score identified IP with a negative predictive value of 0.950 in the low-risk group and a positive predictive value of 1.000 in the high-risk group, whereas the overall area under the curve was 0.998 (95% confidence interval, 0.992–1.000). **Conclusions:** The statistically derived predictive model––ultrasound showing no surrounding endometrium and myometrial thinning < 5 mm combined with vaginal bleeding––demonstrated high accuracy and practical applicability for IP diagnosis, providing a robust tool to enhance clinical decision-making and optimize routine management strategies for IP.

## 1. Introduction

The incidence of interstitial pregnancy (IP) ranges from 1.0% to 6.3% of all ectopic pregnancies and is increasing due to the growing popularity of assisted reproductive technologies [1,2,3]. Most cases are diagnosed surgically following uterine rupture and are managed through cornual resection or hysterectomy [4]. Although high-resolution ultrasound (US) and rapid quantitative β human chorionic gonadotropin (β-hCG) assays have improved early diagnosis, IP remains challenging owing to its location [5,6,7].

Distinguishing IP from an eccentrically located intrauterine pregnancy (IUP) using ultrasound is challenging, as IP is often mistaken for angular or cornual pregnancy [8]. An IP is an ectopic pregnancy implanted in the interstitial portion of the fallopian tube that passes through the uterine muscular wall [9]. An angular pregnancy implies a viable IUP implanted in the cornual region medial to the uterotubal junction. A cornual pregnancy is an IUP located in the rudimentary horn of a unicornuate uterus or the horn of a bicornuate uterus [8]. Overlapping features, such as eccentric GS location and myometrial thinning, make differentiation difficult, often leading to delayed diagnosis and increased risk of uterine rupture [10,11,12]. Given these challenges, early IP identification is crucial for timely intervention [13].

Despite advances in imaging techniques, significant diagnostic challenges remain. The accuracy of 2D transvaginal ultrasound (TVUS) in detecting IP has not been extensively studied [14,15,16,17]. The interstitial line sign [16], an essential diagnostic tool with high sensitivity, is too specific and infrequent; therefore, its clinical application in emergencies is limited.

Given the limitations of current diagnostic techniques, more reliable prediction models for diagnosing IP are needed. These models should integrate clinical, biochemical, and imaging data. Although predictive models for ectopic pregnancy exist, there are currently no widely validated or clinically implemented prediction models specifically designed to diagnose IP [18,19]. Therefore, we aimed to develop and validate a simple scoring-based prediction system to improve the early diagnosis of IP.

## 2. Materials and Methods

### 2.1. Patients

This retrospective cohort study included women with suspected IP who visited a tertiary center between January 2006 and December 2023 to confirm their pregnancy or for a second opinion owing to suspicion of IP based on an eccentrically located GS. Patients with unstable hemodynamics who did not undergo US examination before surgery, those who did not have available US scan images, and those who were lost to follow-up were excluded. The institutional review board of our hospital approved this study (4-2017-0559).

### 2.2. Data Collection and US Examinations

Clinical data, including demographic characteristics; gynecological, obstetric, and surgical history; date of last menstrual period; presenting symptoms (abdominal pain, vaginal bleeding); serum β-hCG levels; treatment methods; operative reports; surgical procedures; and follow-up notes, were collected.

**Figure 1:**
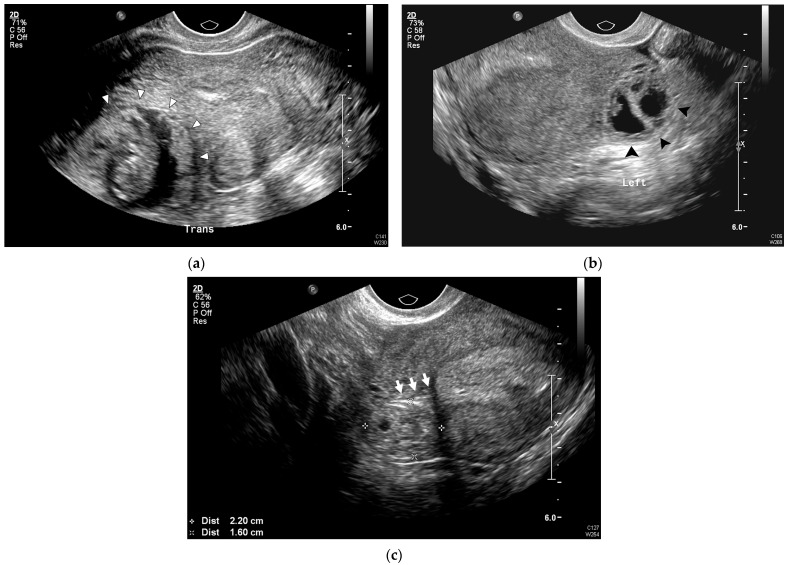
Representative US images of IP. (**a**) Eccentrically located round ring-like mass (white arrowhead) in the right uterine cornu in the transverse plane. (**b**) Asymmetric, thin myometrial (<5 mm) layer surrounding the GS (black arrowhead), without surrounding endometrium. (**c**) Empty uterine cavity and interstitial portion of the tube extending from the uterine cavity to the GS (interstitial line sign, white arrow). GS, gestational sac; IP, interstitial pregnancy; US, ultrasound.

**Figure 2:**
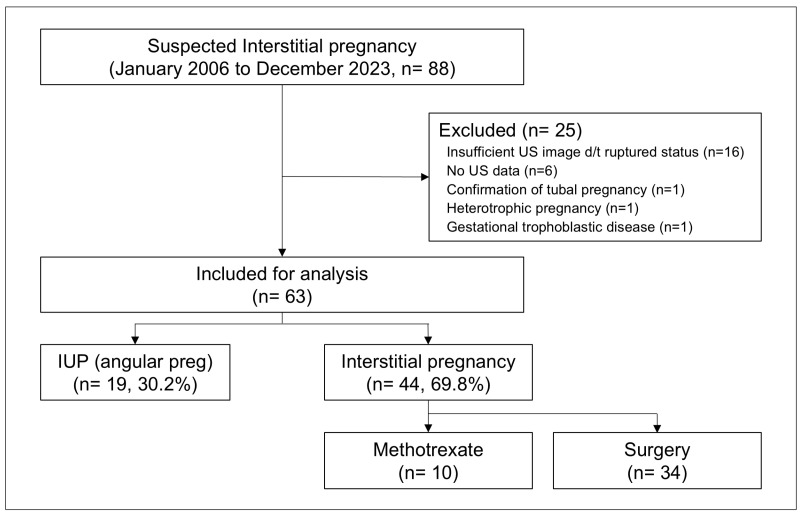
Study population.

**Figure 3:**
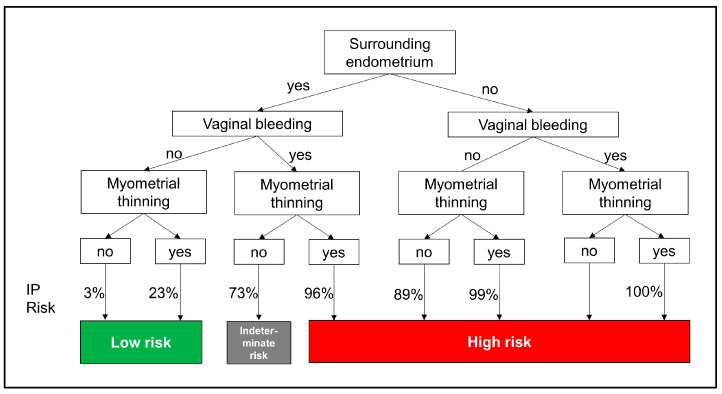
Decision tree model for predicting IP diagnosis.

US examinations were performed using the following ultrasonography systems: Philips iU22 (Philips Healthcare, Bothell, WA, USA), WS80A (Samsung Medison, Seoul, Republic of Korea), and Voluson E10 (GE Healthcare Ultrasound, Milwaukee, WI, USA) with a 5–9 MHz transvaginal transducer. US scans were performed by attending physicians or residents and fellows supervised by attending physicians. The following US findings, characteristic of IP, were extracted from US images [14,15,16,17]: (1) an eccentrically located GS with an empty uterine cavity (Figure 1a); (2) asymmetry of the myometrial mantle around the GS, including myometrial thinning, defined as a myometrial thickness less than 5 mm surrounding the GS (Figure 1b); (3) absence of surrounding endometrium [17], defined as a hyperechogenic endometrial lining encircling the GS (Figure 1b); and (4) presence of the interstitial line sign [16], defined as an echogenic line extending from the endometrium to the cornual region, adjacent to the interstitial mass or GS (Figure 1c).

### 2.3. Statistical Analysis

Descriptive statistics were analyzed, and comparisons by IP status were made using the Mann–Whitney U test for continuous variables and Pearson’s χ^2^ test or Fisher’s exact test for categorical variables. Diagnostic indices, including sensitivity, specificity, and positive and negative predictive values (PPV and NPV, respectively), were calculated to evaluate the accuracy of the TVUS findings for IP diagnosis.

Logistic regression analysis with IP status as the binary outcome was used to develop an IP risk scoring system from which the predicted probabilities of IP could be calculated. Firth-corrected logistic regression [20] addressed sparse data caused by zero-frequency counts in certain combinations of categorical predictors.

Univariable logistic regression results were first examined for predictive feature selection, followed by Akaike Information Criterion (AIC)-based forward, backward, and stepwise selection [21]. The least absolute shrinkage selection operator (LASSO)-based feature importance [22] was also assessed for confirmation and comparison with AIC-based selection [23]. Candidate multivariable models were comparatively evaluated through repeated 5-fold cross-validation (50 repeats each) [24] using the area under the curve (AUC) as the criterion. To develop a practical risk score, we assigned weighted points proportional to the predicted probabilities of the IP (rounded to the nearest integer) derived from the final multivariable model. A risk score was then calculated for each patient, and the population was divided into three risk groups: patients with low, indeterminate, or high risk of IP.

All tests were two-sided, and statistical significance was *p* < 0.05. Analyses were performed using R version 4.3.2 (R Foundation for Statistical Computing, Vienna, Austria).

## 3. Results

During the 17-year study period, 88 patients who visited our hospital were diagnosed with suspected IP. Of these, 16 who could not undergo ultrasonography underwent immediate surgical treatment or were diagnosed with IP during surgery and were excluded from the study. Six were excluded because ultrasound data were unavailable. One patient was diagnosed with tubal pregnancy, another with heterotopic pregnancy, and one patient was diagnosed with gestational trophoblastic disease, which resulted in their exclusion from the study cohort. Finally, 63 patients were included in the analysis. The study population was divided into two groups: patients with a final IUP diagnosis (IUP group, n = 19) and those diagnosed with IP (IP group, n = 44). The patients diagnosed with IP either underwent surgical treatment, with IP confirmed through histopathological examination (n = 34), or conservative medical treatment with systemic administration of methotrexate (n = 10), according to the attending physician’s decision (Figure 2).

### 3.1. Baseline Characteristics and US Findings

Table 1 summarizes the demographic and clinical characteristics of the study population. Compared to the IUP group, the IP group exhibited relatively higher gravidity (*p* = 0.004) and a significantly higher proportion of multiparous women (*p* = 0.042). Vaginal bleeding was significantly more common in the IP group (*p* = 0.020).

Table 2 presents the TVUS findings that differentiated IP from IUP. An eccentrically located GS was more commonly observed in the IP group than in the IUP group. In most cases, the endometrium surrounding the GS was not visible. The mean thickness of the myometrium surrounding the GS was lower in the IP group than in the IUP group, and the rate of myometrial thinning (myometrial thickness < 5 mm) was significantly higher in the IP group than in the IUP group. Furthermore, the interstitial line sign, a reliable diagnostic marker, was observed more frequently in the IP group than in the IUP group, although it did not demonstrate high predictive performance.

### 3.2. Univariable Analysis

We performed univariable (Firth-corrected) logistic regression and receiver operating characteristic (ROC) curve analyses of individual features. Table S1 displays each candidate feature for predicting IP in the univariable analysis with the corresponding AUC values. Gravidity, parity, history of induced abortion, and vaginal bleeding were significantly associated with an increased risk of IP (*p* < 0.05 for all). In terms of the US findings, the presence of surrounding endometrium (odds ratio = 0.003, *p* < 0.001) and increased myometrial thickness (odds ratio = 0.23, *p* < 0.001) were correlated with a decreased risk of IP, whereas the presence of an interstitial line sign (odds ratio = 18.54, *p* = 0.003) was associated with an increased risk of IP.

### 3.3. Selection of the Important Predictors of IP

Predictive variables were selected using AIC-based forward, backward, and stepwise selection methods to identify crucial variables for IP prediction. Among the clinical variables, gravidity, abdominal pain, and vaginal bleeding were important. Among the US findings, the absence of the surrounding endometrium and myometrial thickness were significant predictors. Additionally, LASSO regression was employed to compare with AIC-based selection and prevent model overfitting. The final four predictors identified using the optimal lambda penalty in the LASSO model were the surrounding endometrium, myometrial thickness, vaginal bleeding, and gravidity (Figure S1).

### 3.4. Development and Evaluation of the IP Prediction Model

As shown in Table S2, the absence of the surrounding endometrium, myometrial thinning (<5 mm), vaginal bleeding, and gravidity associated with IP were used to evaluate the predictive ability of the models. The combination of the absence of the surrounding endometrium, myometrial thinning, and vaginal bleeding showed a superior mean AUC (0.992; 95% confidence interval [CI], 0.964–1.000) in terms of repeated five-fold cross-validated predictive performance (50 repeats). Using a five-point scale, a simplified risk score (RS) was developed from the final multivariable prediction model presented in Table 3, as follows.

RS = 5 × (1 − surrE) + 5 × (surrE × mThin × vBleed) + 3.5 × surrE × (1 − mThin) × vBleed + 1 × surrE × mThin × (1 − vBleed), surrE (surrounding endometrium): Yes = 1, No = 0, mThin (myometrial thinning < 5 mm): Yes = 1, No = 0; vBleed (vaginal bleeding): Yes = 1, No = 0.

The RS values ranged from 0 to 5, and Table 4 shows the relationship between the RS and the predicted probabilities. Based on the predicted probabilities for IP, the patients were classified into three risk groups: low-risk (RS: 0–1; predicted probability: 0.03; n = 20), indeterminate-risk (RS: 1–3.5; predicted probability: 0.228–0.733; n =1), and high-risk (RS: 5; predicted probability: 0.892–1.000; n = 42).

Notably, only one patient was classified as indeterminate (predicted probability = 0.733). While they did not meet the high-risk threshold, the probability was too high to rule out IP with confidence, warranting a conservative classification to prioritize patient safety.

The ROC curve analysis (Figure S2) showed an AUC of 0.998 (95% CI, 0.992–1.000), demonstrating the excellent diagnostic performance of the final multivariable IP prediction model-based three-tier scoring system. The low-risk group (RS = 0) had a sensitivity of 97.7% and perfect specificity (100%), with a perfect PPV (100%) and NPV of 95.0%. Conversely, the high-risk group (RS = 5) achieved a sensitivity, specificity, PPV, and NPV of 95.5%, 100%, 100%, and 90.5%, respectively. If a risk score occurs in the low-risk group, it can be interpreted that IP is not possible. If it occurs in the high-risk group, it can be interpreted that IP is almost certain. These results demonstrated the clinical risk stratification ability of the low- and high-risk groups (Table 5).

As shown in Figure 3, this decision tree model provides a structured and intuitive approach for assessing the risk of IP based on key predictors, including the absence of surrounding endometrium, myometrial thinning, and vaginal bleeding. The model simplifies complex diagnostic pathways, demonstrating clinical utility by enabling the early prediction of IP, which may require immediate interventions, including fertility-sparing treatments.

### 3.5. Subgroup Analysis (Surgically Confirmed IP Versus IUP)

A risk remained that cases diagnosed as IP via US and treated with systemic methotrexate treatment may have included cases of misdiagnosed IUP. Therefore, to ensure that the performance of our predictive model remains consistent even when cases with such possibilities are excluded, we performed a subgroup analysis by comparing the IP group confirmed through surgical treatment with the IUP group. The multivariable analysis of this subgroup revealed that gravidity, abdominal pain, and vaginal bleeding were important variables associated with surgically confirmed IP. Among the US findings, the absence of the surrounding endometrium and myometrial thinning were also significantly associated with surgically confirmed IP. The combination of the presence of surrounding endometrium, myometrial thinning, and vaginal bleeding yielded a remarkable mean AUC of 0.990 (95% CI, 0.967–1.000) in terms of the repeated five-fold cross-validated predictive performance (50 repeats). The subgroup analysis also consistently validated the final prediction model based on the five-point scale. Based on the simplified risk score, the patients in the subgroup were also classified into three risk groups. The diagnostic performances of these three risk group classifications demonstrated consistent results, with an AUC of 0.997 (95% CI, 0.990–1.000). In the subgroup analysis, the low-risk group (RS = 0) had a sensitivity of 97.1% and specificity of 100%, with a PPV of 100% and NPV of 95.0%. Conversely, the high-risk group (RS = 5) achieved a sensitivity, specificity, PPV, and NPV of 94.1%, 100%, 100%, and 90.5%, respectively. Reanalyzing the data after excluding the IP cases treated with medical management confirmed that the predictive model derived in this study is reasonable and effective for the diagnosis of IP.

## 4. Discussion

### 4.1. Principal Findings

This study developed a robust and practical scoring system for IP diagnosis using three easily identifiable ultrasound-based predictors. This model provides a superior accuracy and is particularly suited for clinical decision-making in emergent settings where rapid assessment is critical and additional imaging may not be available.

Traditional diagnostic US markers have inconsistent sensitivity and specificity. Timor-Tritsch et al. [15] proposed three US criteria for diagnosing IP—an empty uterine cavity, a separate chorionic sac (>1 cm) from the lateral edge, and a thin myometrial layer (<5 mm)—with sensitivity around 40% (dropping to 25–33% without a demonstrable GS) and specificity ranging from 62% to 93%. Ackerman et al. [16] identified the interstitial line sign as a unique echogenic line in the cornual region, demonstrating 80% sensitivity and 98% specificity, outperforming eccentric GS location and myometrial thinning. However, another study found that 13% of IP cases lacked this sign, highlighting its limitations [17]. The “surrounding endometrium” in the first-trimester US demonstrated good interobserver and intraobserver agreement in distinguishing IP from eccentric IUP, but its reliability remained uncertain due to the small sample size and lack of prospective validation [17]. Rather than evaluating US findings alone, a practical prediction model for IP diagnosis that combines clinical factors is needed.

In this study, we developed a risk stratification model by combining clinical factors and specific US findings associated with IP. The multivariable analysis revealed that gravidity, abdominal pain, and vaginal bleeding were important variables associated with IP. Among the US findings, the absence of the surrounding endometrium and myometrial thinning were significantly associated with IP. Finally, the combination of the absence of surrounding endometrium, myometrial thinning (<5 mm), and vaginal bleeding yielded a remarkable AUC of 0.992. Using a five-point risk score, the patients were classified into three IP risk groups. These results confirm the model’s strong risk stratification ability and excellent diagnostic performance (AUC = 0.998).

### 4.2. Clinical Implications

This study proposed a practical and accessible risk stratification model for diagnosing IP, combining key ultrasound markers with clinical symptoms to improve diagnostic accuracy and facilitate timely decision-making. The three-tier scoring system—comprising low-, indeterminate-, and high-risk categories—was particularly well-suited for emergency settings where rapid assessment was critical and advanced imaging modalities such as 3D ultrasonography or magnetic resonance imaging (MRI) were often unavailable.

Compared to traditional diagnostic markers such as the interstitial line sign [16], which suffered from limited sensitivity and interobserver variability, the model incorporated objective and reproducible features: the absence of surrounding endometrium, myometrial thinning, and vaginal bleeding. This approach enabled consistent diagnostic performance across various clinical environments. Notably, the model extended beyond simple probability estimation by providing a structured risk framework that facilitated nuanced clinical decision-making, particularly in ambiguous cases where conservative management could be appropriate. In our cohort, one patient was classified into the indeterminate-risk group with a predicted probability of 0.733. Although this value did not meet the high-risk threshold, it was too high to confidently exclude IP. In such cases, the model supported a conservative approach—recommending further imaging or close monitoring—to minimize diagnostic error and prioritize patient safety. This structured, risk-based strategy allowed clinicians to navigate uncertainty and avoid both over- and under-treatment in complex presentations. Nonetheless, the rarity of indeterminate cases in this cohort warrants cautious interpretation regarding the clinical utility of this category.

The decision tree derived from the model provides a highly interpretable visual tool for bedside application. As shown in Figure 3, the model sequentially evaluates the absence of surrounding endometrium, myometrial thinning (<5 mm), and vaginal bleeding—each representing pathophysiologically relevant diagnostic markers. The absence of surrounding endometrium suggests extra-endometrial implantation, myometrial thinning indicates an increased risk of uterine rupture, and vaginal bleeding may reflect pregnancy instability. This hierarchical logic mirrors real-world clinical reasoning and supports intuitive decision-making without the need for complex computation. The transparency of the decision tree fosters clinician trust and facilitates implementation in routine workflows, particularly in time-sensitive or resource-limited settings.

Furthermore, the model demonstrated potential utility in identifying appropriate candidates for conservative medical treatment, which emerged as a promising alternative to surgery—particularly for patients desiring fertility preservation. Previous studies emphasized that early diagnosis in hemodynamically stable patients with low serum β-hCG levels and early gestational age could enable the effective use of methotrexate, either alone or in combination with mifepristone [25]. By facilitating early and accurate risk stratification, the model provided a framework to support such fertility-sparing strategies before complications necessitated invasive intervention.

### 4.3. Research Implications

Our study introduces the first predictive model for IP, addressing a gap where previous models focused only on ectopic pregnancy or pregnancy of unknown location (PUL). Predictive analytical models using clinical factors and ultrasound findings to predict abnormal pregnancy are becoming more advanced, with the application of logistic regression models from M1 to M6 and machine learning development [26]. The M4 prediction model, which utilizes initial serum hCG levels and hCG ratios, demonstrated an AUC of 0.84 for predicting ectopic pregnancy [27]. The M6 model, which enhanced the M4 model by incorporating progesterone levels, achieved an improved performance with an AUC of 0.94 [28]. Rueangket et al. used neural networks, decision trees, support vector machines, and statistical logistic regression analyses of 22 study features based on the clinical factors of PUL, serum markers, and ultrasound findings from electronic medical records. The average performance was high in all models (AUC ≥ 0.856) [19]. Despite promising research outcomes, these models lack clinical implementation, and no predictive tool for IP has been developed, underscoring the novelty and impact of our model.

### 4.4. Strengths and Limitations

This study presents a novel, high-performance predictive model for early IP diagnosis, addressing a key gap in clinical practice. Using robust statistical methods, we developed a highly accurate scoring system (AUC = 0.998) with strong sensitivity and specificity. A major strength is that it offers a practical, resource-efficient tool for rapid decision-making in emergency settings, where timely diagnosis is crucial. Additionally, our rigorous validation, including a subgroup analysis excluding medically managed cases, confirmed the model’s robustness and clinical applicability, supporting its real-world implementation.

Despite these strengths, several limitations should be acknowledged. First, this was a retrospective, single-center study, which may introduce selection bias and limit the generalizability of our findings. In particular, only one patient in our cohort was classified into the indeterminate risk group, which limited our ability to evaluate the clinical utility of this category. To address this limitation, future research should involve a large-scale, multicenter prospective cohort study—ideally including surgically confirmed cases—to test the model’s applicability across diverse clinical settings and to refine cutoff thresholds for indeterminate classification. Second, while our model focuses on 2D ultrasound for accessibility, it does not incorporate 3D US imaging, which may enhance the diagnostic precision by providing better spatial mapping of the pregnancy within the myometrium [29,30,31]. However, 3D/4D US studies are mostly case reports and evidence on their diagnostic yield remains limited [32,33,34]. In emergency settings, point-of-care ultrasound remains more practical than 3D volume acquisition and rendering [35]. We believe that developing a simple 2D-based scoring system will be beneficial for managing critical complications. Third, interobserver variability in ultrasound interpretation may affect consistency. This highlights the need for automated image analysis or artificial intelligence-assisted interpretation in future research.

## 5. Conclusions

In conclusion, our model is a simple and powerful risk stratification tool for accurately predicting the IP risk. It enhances clinical decision-making and streamlines management strategies with high diagnostic accuracy and practical applicability. This robust predictive framework may provide a reliable approach to improving patient outcomes and optimizing routine care.

## Figures and Tables

**Table 1:** Demographics and clinical characteristics of the study population.

Variables	IUP (n = 19)	IP (n = 44)	*p*-Value
Maternal age (years)	31.2 ± 7.0	32.1 ± 4.6	0.628
Gravida	1.7 ± 0.9	2.7 ± 1.4	0.004
Nullipara	16 (84.2)	23 (52.3)	0.042
Previous history of induced abortion	5 (26.3)	25 (56.8)	0.051
Previous history of ectopic pregnancy	2 (10.5)	10 (22.7)	0.434
Previous history of tubal surgery	0	3 (6.8)	0.602
In vitro fertilization	0	6 (13.6)	0.240
Clinical manifestations			
Vaginal bleeding	0	13 (29.5)	0.020
Abdominal pain	1 (5.3)	11 (25.0)	0.138
Serum β-hCG (mIU/mL)	35610.9 ± 37610.4	31237.2 ± 36365.8	0.759

Values are presented as n (%) or mean ± standard deviation (SD). IP, interstitial ectopic pregnancy; IUP, intrauterine pregnancy.

**Table 2:** Ultrasound characteristics in the study population.

Ultrasound Findings	IUP (n = 19)	IP (n = 44)	*p*-Value
Mean sac diameter at diagnosis (mm)	22.9 ± 8.6	24.5 ± 8.8	0.516
Eccentrically located GS	15 (78.9)	44 (100.0)	0.010
Absent surrounding endometrium	0 (0.0)	40 (90.9)	<0.001
Myometrial thickness			
Myometrial thickness (mm)	6.7 ± 2.5	2.6 ± 1.2	<0.001
Myometrial thinning (<5 mm)	3 (15.8)	41 (93.2)	<0.001
Interstitial line sign	0 (0.0)	14 (31.8)	0.014

Values are presented as n (%) or mean ± standard deviation (SD). GS, gestational sac; IP, interstitial ectopic pregnancy; IUP, intrauterine pregnancy.

**Table 3:** The final multivariable model for IP prediction.

Variable	OR (95% CI)	*p*-Value
(Intercept)	8.23 (1.29–4.74)	0.157
Surrounding endometrium	0.004 (0.000–0.05)	<0.001
Myometrial thinning (<5 mm)	9.54 (0.45–1547)	0.146
Vaginal bleeding	88.5 (3.61–39775)	0.004

OR, odds ratio; IP, interstitial pregnancy; CI, confidence interval.

**Table 4:** The predicted probabilities of IP from the final multivariable model and their corresponding 5-point scale risk scores for IP prediction.

Variable Conditions				Predicted Probability	5-Point Scale	Risk Group
Surrounding Endometrium	Vaginal Bleeding	Myometrial Thinning	N			
Yes	No	No	15	0.030	0	Low
Yes	No	Yes	5	0.228	1	
Yes	Yes	No	1	0.733	3.5	Indeterminate
Yes	Yes	Yes	2	0.963	5	High
No	No	No	2	0.892	5	
No	No	Yes	28	0.987	5	
No	Yes	Yes	9	1.000	5	

IP, interstitial pregnancy.

**Table 5:** Clinical risk stratification performance of three-tier risk groups for IP prediction.

(**a**) IP = Yes/No cross-tabulations based on three-tier risk groups					
Risk Groups		IP = No		IP = Yes	
Low-risk		19		1	
Indeterminate		0		1	
High-risk		0		42	
(**b**) Diagnostic performance for IP prediction based on three-tier risk groups					
Risk Groups	Sensitivity	Specificity	PPV	NPV	AUC (95% CI)
Low-risk	97.7%	100%	100%	95.0%	0.998 (0.992–1.000)
Indeterminate	-	-	-	-	
High-risk	95.5%	100%	100%	90.5%	

IP, interstitial pregnancy; AUC, area under the curve; CI, confidence interval; PPV, positive predictive value; NPV, negative predictive value.

## Data Availability

The datasets collected and/or analyzed during the current study are available from the corresponding author upon reasonable request.

## Supplementary Materials

The following supporting information can be downloaded at https://www.mdpi.com/article/10.3390/jcm14124238/s1, Table S1. Odds ratios estimated from univariable logistic regression analysis and corresponding area under the curve estimated from receiver operating characteristic analysis for IP prediction in relation to clinical and ultrasound findings; Table S2. Comparison of mean area under the ROC curves of repeated 5-fold cross-validations (50 repeats each) among the candidate multivariable models for IP prediction; Figure S1. Feature selection by LASSO regression. Lasso regression graph of the clinical predictors; Figure S2. The receiver operator characteristic (ROC) curve for the predictive risk scoring model.

## Abbreviations

The following abbreviations are used in this manuscript:

LASSO	Least absolute shrinkage selection operator
AIC	Akaike Information Criterion
IP	Interstitial pregnancy
IUP	Intrauterine pregnancy
β-hCG	β human chorionic gonadotropin
US	Ultrasound
TVUS	2D transvaginal ultrasound
PPV	Positive predictive value
AUC	Area under the curve
NPV	Negative predictive value
ROC	Receiver operating characteristic
