# Supplementary material for: The Development of an Ultrasound-Based Scoring System for the Prediction of Interstitial Pregnancy
Source: J Clin Med. 2025 Jun 14;14(12):4238. doi: 10.3390/jcm14124238 (PMC12194485; doi:10.3390/jcm14124238)
Supplement: Supplementary file 1 [file jcm-14-04238-s001.zip › jcm-3686132-supplementary.pdf]

*Article*

# The Development of an Ultrasound-Based Scoring System for the Prediction of Interstitial Pregnancy

## Supplementary Information

### 1. Supplementary Tables

**Table S1.** Odds ratios estimated from univariable logistic regression analysis and corresponding area under the curve estimated from receiver operating characteristic analysis for IP prediction in relation to clinical and ultrasound findings.

| **Variable** | **OR (95% CI)** | ***p*-value** | **AUC (95% CI)** |
|---|---|---|---|
| Gravidity | 2.27 (1.29–4.74) | 0.002 | 0.734 (0.606–0.862) |
| Parity | 3.67 (1.32–14.4) | 0.010 | 0.670 (0.563–0.777) |
| Prior induced abortion history | 3.46 (1.14–11.6) | 0.027 | 0.652 (0.527–0.778) |
| Prior ectopic history | 2.14 (0.54–12.0) | 0.297 | 0.561 (0.466–0.656) |
| Abdominal pain | 4.22 (0.89–41.2) | 0.072 | 0.599 (0.516–0.681) |
| Vaginal bleeding | 16.78 (2.01–2182) | 0.004 | 0.648 (0.579–0.716) |
| Mean sac diameter | 1.02 (0.96–1.09) | 0.517 | 0.563 (0.407–0.720) |
| Eccentric GS | 25.79 (2.53–3498) | 0.004 | 0.605 (0.511–0.699) |
| Surrounding endometrium | 0.003 (0.00–0.03) | <0.001 | 0.954 (0.912–0.997) |
| Myometrial thickness | 0.23 (0.07–0.47) | <0.001 | 0.947 (0.896–0.999) |
| Interstitial line sign | 18.54 (2.24–2419) | 0.003 | 0.659 (0.589–0.729) |

OR, odds ratio; AUC, area under the curve; ROC, receiver operating characteristic; IP, interstitial ectopic pregnancy; CI, confidence interval; GS, gestational sac

**Table S2.** Comparison of mean area under the ROC curves of repeated 5-fold cross validations (50 repeats each) among the candidate multivariable models for IP prediction.

| **Model** | **AUC (95% CI)** |
|---|---|
| Surrounding endometrium + myometrial thickness | 0.985 (0.975–0.999) |
| Surrounding endometrium + vaginal bleeding | 0.985 (0.971–1.000) |
| Surrounding endometrium + vaginal bleeding + gravida | 0.993 (0.926–0.991) |
| Myometrial thinning (<5 mm) + interstitial sign + vaginal bleeding | 0.938 (0.912–0.960) |
| Surrounding endometrium + myometrial thinning (<5 mm) + vaginal bleeding | 0.992 (0.964–1.000) |

AUC, area under the curve; ROC, receiver operating characteristic; IP, interstitial pregnancy; CI, confidence interval

## 2. Supplementary Figures

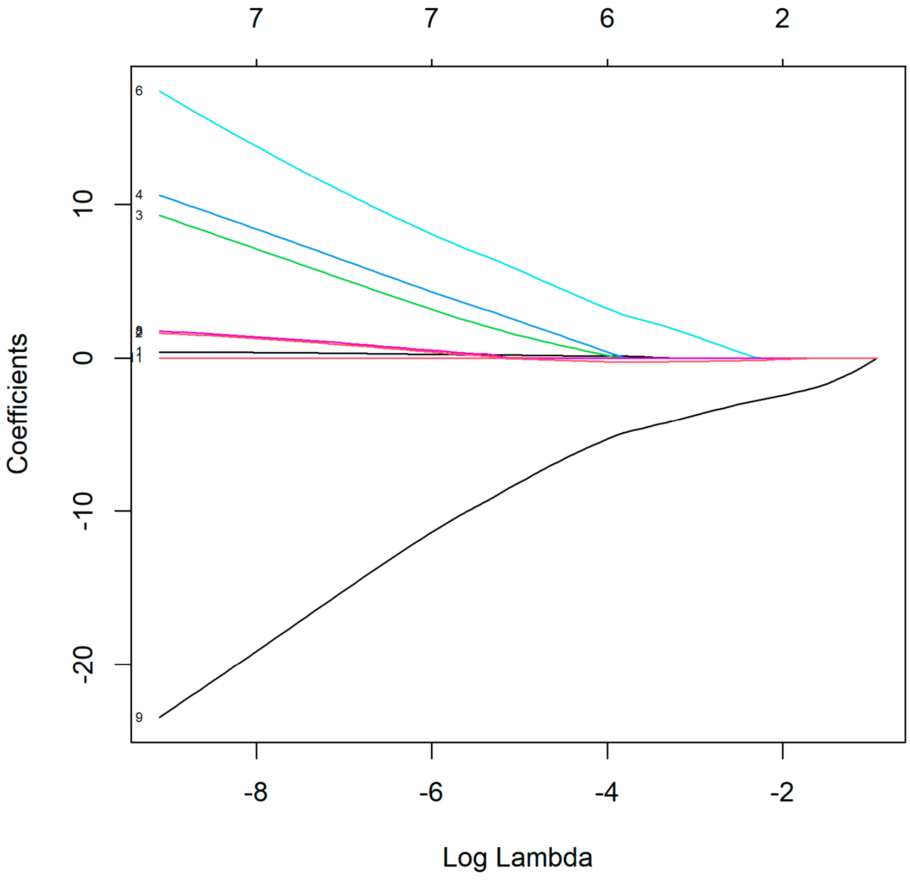


**Figure S1.** Feature selection by LASSO regression. Lasso regression graph of the clinical predictors

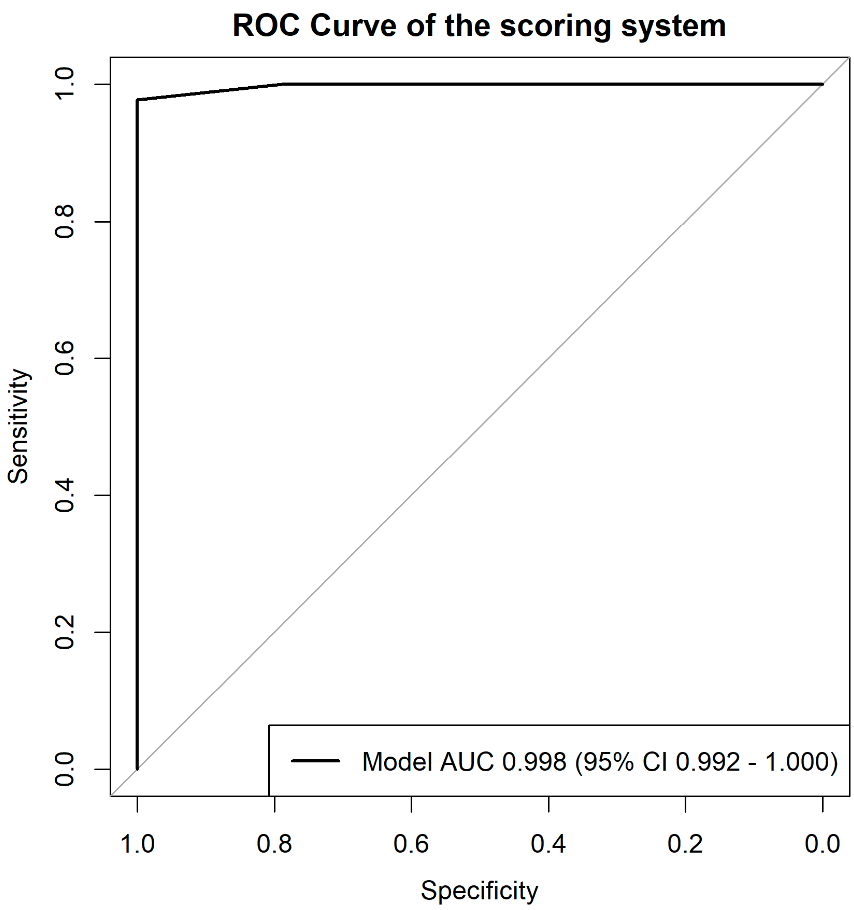


**Figure S2.** The receiver operator characteristics (ROC) curve for the predictive risk scoring model.
